# Occupational stress, burnout, and depression: an exploration from a network analysis perspective

**DOI:** 10.1017/S0033291726103547

**Published:** 2026-04-27

**Authors:** Yiyuan Qiao, Haitao Xu, Yuling Li, Lei Shi, Shiqian Zhen, Shuchang He, XiangYang Zhang

**Affiliations:** 1 School of Psychological and Cognitive Sciences and Beijing Key Laboratory of Behavior and Mental Health; Key Laboratory of Machine Perception (Ministry of Education), Peking University, 5 Yiheyuan Road, Haidian District, Beijing 100871, China; 2 Key Laboratory of Brain, Cognition and Education Sciences, Ministry of Education; Institute for Brain Research and Rehabilitation, and Guangdong Key Laboratory of Mental Health and Cognitive Science, South China Normal University, Guangzhou 510631, China; 3 Department of Medical Research, The Ninth Medical Center, Chinese PLA General Hospital, Beijing 100101, China; 4 Institute of Circulation and Consumption, Chinese Academy of International Trade and Economic Cooperation, 28 Donghou Lane, Andingmenwai, Dongcheng District, Beijing 100710, China; 5 Department of Psychological and Cognitive Sciences, Tsinghua University, Haidian District, Beijing 100084, China

**Keywords:** burnout, depression, network analysis, occupational stress

## Abstract

**Background:**

Occupational stress triggers psychological/physical health issues, elevating the risk of burnout and depression. This study explored the interrelationships among these constructs via network analysis (undirected/directed graphs).

**Methods:**

A total of 1363 participants from Beijing hospitals and a university completed House and Rizzo’s Work Stress Scale, Zung’s Self-Report Depression Scale, and Maslach Burnout Inventory-General Survey. Graphical Gaussian Model and directed acyclic graphs (DAG) identified core/bridge/upstream nodes and causal pathways.

**Results:**

Emotional exhaustion (EE) was the core node (expected influence = 2.11). The strongest edge was D11–D12 (weight = 0.46). EE, occupational stress 11, cynicism (CY), and personal accomplishment (PA) served as key bridging nodes. The network showed high stability (0.75). DAG identified upstream occupational stress 1/7/8, confirming direct occupational stress to depression pathways (emotional dysregulation model) and CY/PA mediated pathways (burnout structural theory).

**Conclusions:**

Targeted interventions on core/bridge/upstream nodes may prevent depression onset and progression in occupational settings.

## Introduction

Occupational stress arises from a detrimental mismatch between the individual and the work environment. When job demands exceed an employee’s current knowledge, skills, or capabilities, occupational stress is triggered. This leads to an inability to cope effectively, subsequently causing psychological and physiological health issues (Folkman, 2013). A growing body of evidence indicates that occupational stress is frequently associated with metabolic syndrome (Chandola, Brunner, & Marmot, 2006), cardiovascular disease (Eddy, Wertheim, Kingsley, & Wright, 2017; Kivimäki et al., 2006), stroke (Pega et al., 2021), cancer (Yang et al., 2018), anxiety (Melchior et al., 2007), occupational burnout (Jia et al., 2021), and depression (du Prel et al., 2024; Oenning, Ziegelmann, Goulart, & Niedhammer, 2018).

According to the Job Demands–Resources (JD-R) model (Bakker & Demerouti, 2007;Bakker, Demerouti, & Sanz-Vergel, 2023), occupational burnout occurs when employees persistently face work demands without sufficient resources to cope or mitigate them. Burnout is defined as a chronic response to occupational stressors, including excessive workload, interpersonal conflict, and emotional strain, characterized by three core dimensions: emotional exhaustion, cynicism, and reduced personal accomplishment (Maslach, Schaufeli, & Leiter, 2001). This three-dimensional model is also proposed to exhibit sequential developmental characteristics (Maslach & Leiter, 2016): Emotional exhaustion emerges first, followed by cynicism, and ultimately leads to reduced personal accomplishment.

Previous studies have examined the conceptual overlap between job burnout and depression among teachers in Australia (Sowden, Schonfeld, & Bianchi, 2022) and the United States (Schonfeld & Bianchi, 2015). Furthermore, multiple investigations have confirmed a significant positive correlation between depression and job burnout, particularly in its affective dimension (Bianchi & Brisson, 2017; Glass, McKnight, & Valdimarsdottir, 1993; Yilmaz, 2018). Similar to burnout, occupational stress is widely recognized as a major contributor to depression. A recent review highlights a significant association between occupational stress and depression (du Prel et al., 2024). This association has been validated across diverse occupational groups, including gas station employees (Tao et al., 2024), teachers (Allam & Desouky, 2017), and healthcare workers (He et al., 2018; H. S. Lin, Probst, & Hsu, 2010). A meta-analysis found that increased occupational stress elevates depression incidence (Lu et al., 2018). Large-scale empirical studies similarly reveal occupational stress as a risk factor for the onset and progression of depressive symptoms (Lam et al., 2013; Melchior et al., 2007). Beyond examining the pairwise relationships among these variables (occupational stress, burnout, and depression), numerous studies have investigated their triadic associations. Complex interactions exist between them. Occupational stress can independently induce both burnout and depression (Iacovides, Fountoulakis, Kaprinis, & Kaprinis, 2003). A prospective study examining the intricate relationship among these three factors yielded two key findings (Ahola & Hakanen, 2007). First, burnout and depression exhibit a bidirectional association. Second, occupational stress leads to depression through the full mediating effect of burnout. Consistent with these findings, burnout was also found to fully mediate the relationship between occupational stress and depression among Chinese teachers (Zhong et al., 2009). However, multiple studies indicate that burnout only partially mediates the relationship between occupational stress and depression, with occupational stress simultaneously acting as another direct influencing factor (T. C. Lin et al., 2016; Steinhardt, Smith Jaggars, Faulk, & Gloria, 2011). From a biological perspective, DNA methylation is key to revealing the underlying mechanisms of this triadic relationship (Bakusic, Schaufeli, Claes, & Godderis, 2017).

The interaction between occupational stress, burnout, and depression is typically analyzed in traditional studies by calculating total scores, rather than focusing on the interplay between symptoms and their shared associations. Emerging network analysis methods can dissect the interconnectedness among symptoms, uncover previously hidden relationships, and identify centrality metrics, thereby providing targets for clinical interventions (Borsboom & Cramer, 2013; McNally, 2016). Network analysis explores interactions through nodes and edges, where nodes represent symptoms and edges denote associations. Centrality measures – including strength (number of nodes directly connected to a specific node), closeness (sum of shortest paths from a node to all others), and betweenness (frequency of a node appearing on shortest paths between any two nodes) – assess an individual node’s importance within the overall network (Epskamp & Fried, 2018). Bridge symptoms aim to connect two psychiatric syndromes (Jones, Ma, & McNally, 2019). Core nodes and bridge symptoms enhance the precision of interventions and treatment plans (Castro et al., 2019). Another network analysis method is directed acyclic graphs (DAGs), which leverage Bayesian inference to explore causal relationships between nodes (Beattie et al., 2023; McNally et al., 2017a, 2017b). DAGs address limitations of undirected network analysis in identifying potential causal relationships between variables (Liu et al., 2025). Arrows denote direct causal relationships between two variables independent of others, clearly illustrating causal order (Feeney, Hartwig, & Davies, 2025).

To our best knowledge, few studies have employed network analysis to examine the relationship between occupational stress, burnout, and depression (Ernst et al., 2021; Verkuilen, Bianchi, Schonfeld, & Laurent, 2020; Zhang Y. et al., 2024), and even fewer comprehensively examined the occupational stress–burnout–depression network. Therefore, this study aimed to employ network analysis techniques to explore this complex association deeply. First, an undirected network was constructed to investigate core and bridging symptoms. Subsequently, DAGs were used to identify potential causal relationships among symptoms, providing a theoretical basis for future intervention measures.

## Methods

### Participants

Initially, 1363 participants from a general hospital and a university in Beijing completed the assessment. After excluding incomplete questionnaires, 772 participants were retained (359 males, 413 females; age range 18–68 years, mean age 36.80 years, standard deviation 10.00). The sample was divided into two groups: hospital medical staff (*n* = 259, 100 males and 159 females; age range 18–62 years, mean age 31.80 years, standard deviation 9.22) and university faculty (*n* = 513, 259 males and 254 females; age range 21–68 years, mean age 39.33 years, standard deviation 9.24).

This study was approved by the Ethics Review Committee of Peking University. All participants signed written informed consent forms in accordance with the requirements of the Declaration of Helsinki.

### Measurement methods

Occupational stress was assessed using the Chinese version of the House and Rizzo’s Work Stress Scale (House & Rizzo, 1972). This scale comprises 11 items, each scored on a 1- to 6-point scale, yielding a total score ranging from 11 to 66 points. Widely applied in China, the scale demonstrates good validity and reliability. Higher scores indicate greater occupational stress levels. In this study, the scale’s alpha coefficient was 0.92.

Depressive symptoms were assessed using the Chinese version of Zung’s Self-Rating Depression Scale (Zung, 1965). This questionnaire comprises 20 items scored on a 4-point Likert scale, where 1 represents ‘never’ or ‘rarely’ and 4 means ‘always’. The total score ranges from 20 to 80, with higher scores indicating more severe depressive symptoms. The alpha coefficient for this scale in this study was 0.84.

Occupational burnout levels were assessed using the Maslach Burnout Inventory-General Survey (MBI-GS) (Maslach & Jackson, 1981). This scale comprises three dimensions: Emotional Exhaustion (EE), Cynicism (CY), and Reduced Professional Accomplishment (PA). It contains 15 items, each scored from 0 (never) to 6 (daily). Higher scores indicate greater burnout. Network analysis and directed acyclic graphs (DAGs) used burnout dimensions rather than individual indicators to clarify mediating pathways. The alpha coefficient for this scale in this study was 0.87.

### Statistical analysis

A cross-sectional network was constructed using R software version 4.5.0, where nodes represented occupational stress, depression, and occupational burnout. We employed a graphical Gaussian model (GGM) combined with the extended Bayesian information criterion (EBIC) to minimize edge counts and eliminate weak connections, thereby constructing an optimal network with the minimum necessary edges (Epskamp & Fried, 2018). The gamma parameter was set to 0.5. The resulting undirected network included nodes and edges representing psychological constructs, along with normalized partial correlation coefficients between constructs. Red edges indicate negative correlations, while blue edges denote positive correlations.

Centrality measures (including strength, closeness, betweenness, and expected influence EI) were computed using the qgraph package, which also provided visualization (Epskamp et al., 2012). Node centrality differences and edge significance were assessed via the bootnet package (Epskamp & Fried, 2018).

We also employ ‘Networktools’ to estimate a bridge’s expected impact, a metric that reveals edges between a symptom originating from one community and a node within another community (Jones et al., 2019). Network reliability was assessed using two methods via the bootnet package: First, bootstrap analysis evaluated edge weight precision through 95% confidence intervals, with narrower intervals indicating higher accuracy. Second, we calculated the correlation stability (CS) coefficient via case-dropping procedures. This coefficient determines the threshold at which, after removing the maximum proportion of cases at the 95% confidence level, the correlation with the original centrality metric remains at 0.7 or higher. A CS coefficient above 0.25 is considered acceptable, while exceeding 0.5 indicates excellent stability. Network comparison test (NCT) analyzed the network characteristics of occupational and gender subgroups by examining global network strength, network structure, and edge strength. Global network strength assessment determined whether total connectivity differed between subgroups; network structure comparison evaluated whether edge weights were consistent across networks; edge invariance testing verified whether specific edges maintained stable weights across different networks (van Borkulo et al., 2023).

Bayesian networks were generated using the hill-climbing algorithm from the bnlearn package (Scutari, 2010) to create DAGs. The Bayesian information criterion (BIC) was optimized through a bootstrap procedure, involving adding/removing edges and adjusting edge directions. We performed 1000 bootstrap samples and averaged the results to determine the final network. Edges appearing in over 85% of networks were retained according to established criteria (Sachs et al., 2005). Additionally, when the direction of an edge from variable X to variable Y appeared in more than 51% of iterations that direction was incorporated into the final graph (McNally et al., 2017a, 2017b, 2022; Sachs et al., 2005). For instance, edge thickness reflected the probability of directed connections: edges appearing in 95% of bootstrap iterations are depicted with thick lines, while those presented in only 51% of samples were shown with thin lines, indicating lower consistency across iterations.

## Results

### Network structure

**Figure 1.**
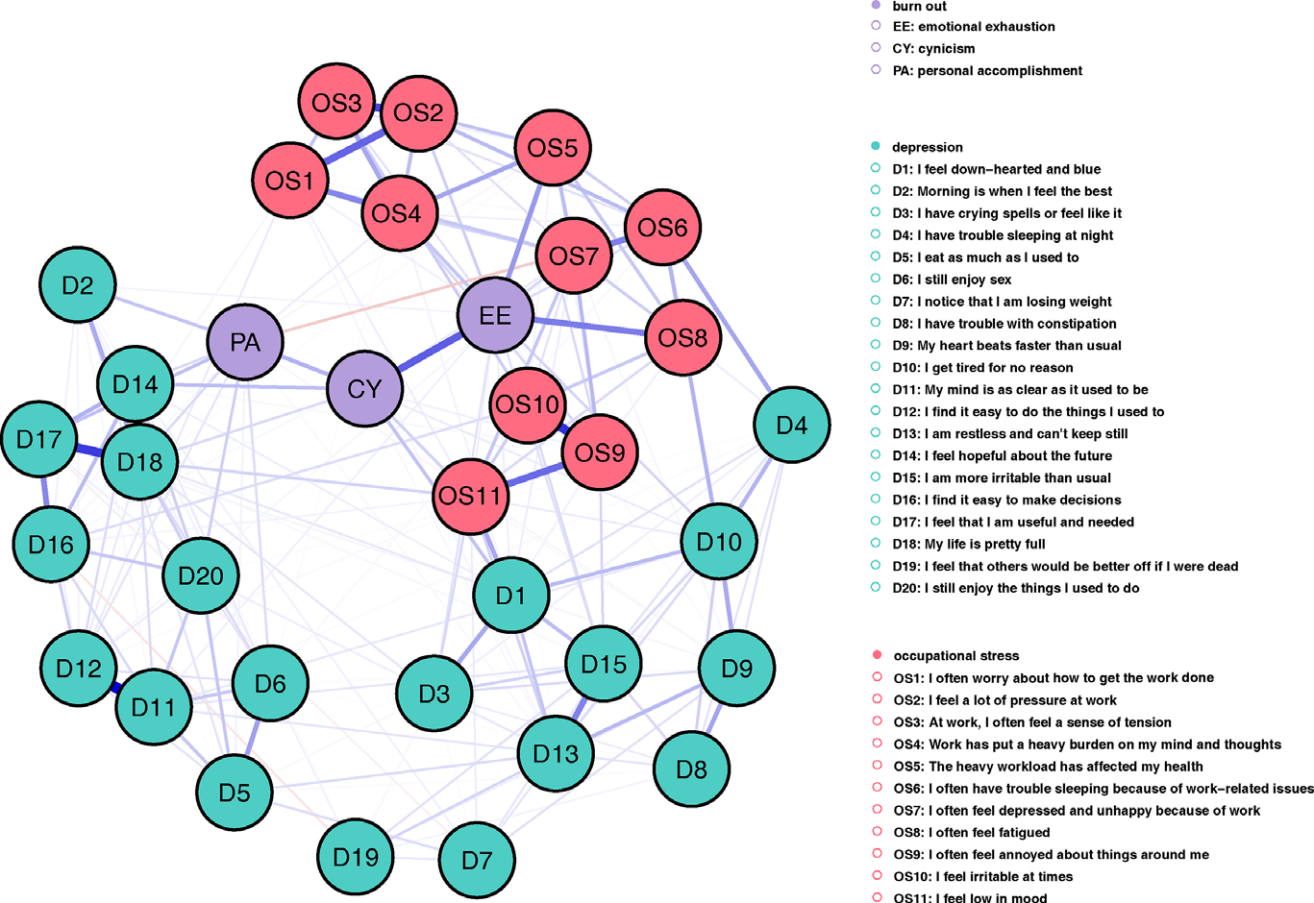
Network of depressive, burnout symptoms and occupational stress among populations with high pressure. *Note*: Nodes in the same color represent the same symptom cluster: green for depressive symptoms, pink for occupational stress, purple for three dimensions from burnout assessment. Thicker edges indicate strong correlations; blue edges indicate positive correlation; and red edges indicate negative correlation.

The occupational stress–burnout–depression network comprised 34 nodes, each representing an item from the overall scale. This network generated 561 possible edges, with 210 nonzero edges, achieving an edge density of 37.4%. Additionally, 97% of edges were positive. The strongest edges occurred within each symptom community (Figure 1): Edge weight between D11 (My mind is as clear as it used to be) and D12 (I find it easy to do the things I used to) reached 0.46; between OS9 (I often feel annoyed about things around me) and OS10 (I feel irritable at times) was at 0.37; D17 (I feel that I am useful and needed) and D18 (My life is pretty full) was at 0.36; OS2 (I feel a lot of pressure at work) and OS3 (At work, I often feel a sense of tension) was at 0.33; EE (Emotional Exhaustion) and CY (Cynicism) was at 0.29.

### Network centrality and bridge centrality

**Figure 2.**
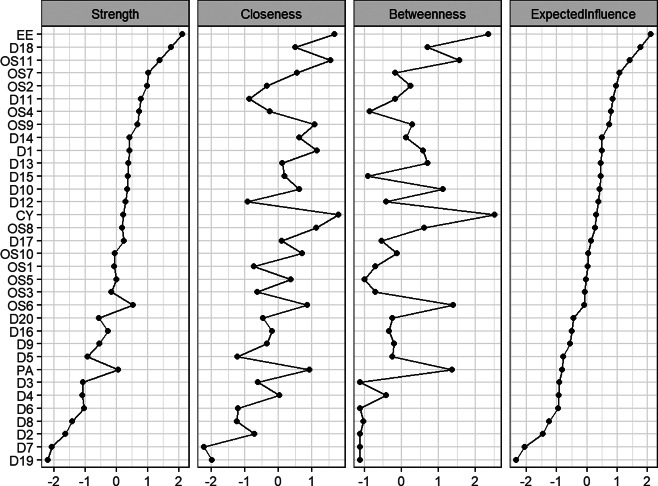
Strength, closeness, betweenness, and expected influence for each node of the network (ranked by *z* scores).

**Figure 3.**
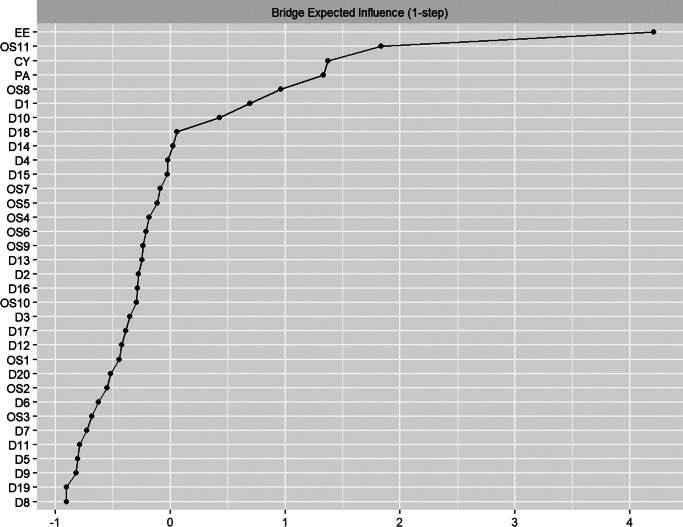
BEI for each node of the network (ranked by *z* scores).

Centrality metrics for each node in the network are detailed in Figure 2 (More specifically in Table 1). Core nodes included EE (Emotional Exhaustion), D18 (My life is pretty full), OS11 (I feel low in mood), and OS7 (I often feel depressed or unhappy because of work), with respective Expected Influence (EI) values of 2.11, 1.76, 1.42, and 1.07. Bridge expected influence is illustrated in Figure 3, where EE (Emotional Exhaustion, BEI = 4.21), OS11 (Low Mood, BEI = 1.84), CY (Cynicism, BEI = 1.37), and PA (Personal Accomplishment, BEI = 1.33) exhibited the highest BEI values.

**Table 1.** The *z* scores of centrality indices

	Expected influence	Strength	Closeness	Betweenness
OS1	0.03	−0.06	−0.73	−0.70
OS2	0.96	0.98	−0.34	0.25
OS3	−0.06	−0.16	−0.63	−0.70
OS4	0.79	0.73	−0.26	−0.86
OS5	−0.04	0.00	0.37	−0.99
OS6	−0.09	0.54	0.86	1.40
OS7	1.07	1.03	0.55	−0.16
OS8	0.27	0.19	1.13	0.62
OS9	0.73	0.67	1.08	0.29
OS10	0.05	−0.05	0.71	−0.12
OS11	1.42	1.39	1.56	1.57
EE	2.11	2.12	1.66	2.35
CY	0.31	0.23	1.79	2.52
PA	−0.82	0.06	0.92	1.36
D1	0.49	0.42	1.15	0.58
D2	−1.45	−1.62	−0.72	−1.11
D3	−0.91	−1.06	−0.61	−1.11
D4	−0.94	−1.08	0.02	−0.41
D5	−0.79	−0.92	−1.22	−0.25
D6	−0.95	−1.03	−1.21	−1.11
D7	−2.07	−2.06	−2.21	−1.11
D8	−1.25	−1.42	−1.23	−1.03
D9	−0.56	−0.54	−0.35	−0.20
D10	0.42	0.35	0.62	1.11
D11	0.85	0.80	−0.86	−0.16
D12	0.38	0.30	−0.91	−0.41
D13	0.46	0.39	0.12	0.70
D14	0.50	0.43	0.62	0.12
D15	0.45	0.37	0.19	−0.91
D16	−0.50	−0.26	−0.20	−0.33
D17	0.13	0.25	0.09	−0.53
D18	1.76	1.76	0.51	0.70
D19	−2.33	−2.18	−1.98	−1.11
D20	−0.44	−0.56	−0.47	−0.25

### Network stability and accuracy

Supplementary Figure S1 presents the results of EI’s nonparametric bootstrap test. The bootstrap results for edge weights showed relatively narrow 95% CI, indicating high network accuracy (Supplementary Figure S2). Both EI and BEI yielded cs-coefficients of 0.75, demonstrating high network stability (Supplementary Figure S3).

### Network comparison tests

Network comparisons between teacher and healthcare worker subgroups revealed no statistically significant differences in global strength (teachers: 14.47, healthcare workers: 12.83, *S* = 1.65, *P* = 0.10) or network structure (*M* = 0.20, *P* = 0.35). Network comparisons between gender subgroups revealed no statistically significant differences in global strength (males: 14.36, females: 13.92, *S* = 0.44, *P* = 0.50) or network structure (*M* = 0.18, *P* = 0.49).

### DAG results

**Figure 4.**
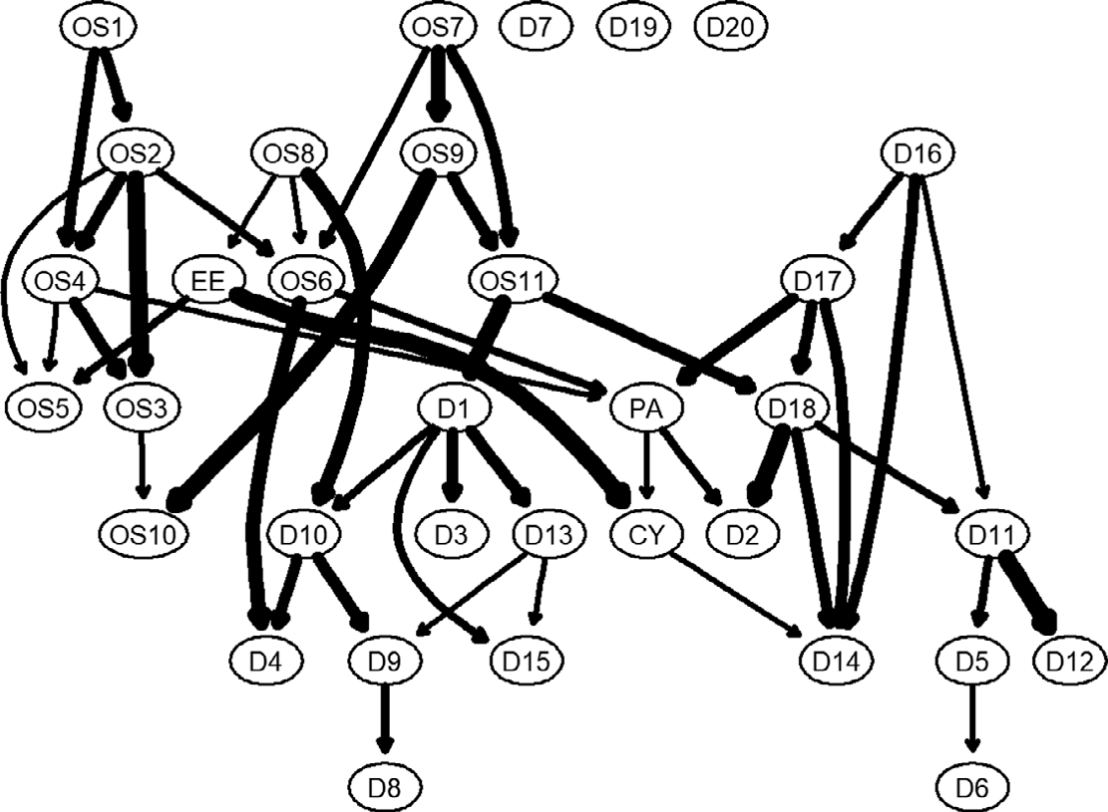
A Bayesian network (directed acyclic graph; DAG) depicting occupational stress–burnout and depression.

In Figure 4, parent nodes primarily originate from the occupational stress cluster: OS1 – ‘I often worry about how to get the work done’, OS8 – ‘I often feel fatigued’, and OS7 – ‘I often feel depressed and unhappy because of work’. These clusters directly predicted other occupational symptoms and some depressive symptoms. Particular attention should be paid to the importance of PA and CY as bridging symptoms within the burnout cluster. They formed a pathway from occupational stress to burnout and ultimately to depression. Crucially, EE did not directly influence depression but first acted upon CY, which finally affected depression.

## Discussion

To our knowledge, this study is the first to employ both undirected and directed network analysis methods to explore the associations among occupational stress, burnout, and depression. First, we identified core and bridging symptoms within this network and analyzed occupational and gender differences. Subsequently, DAGs revealed potential causal relationships among symptoms.

Among depression indicators, the strongest association existed between D11 (‘My mind is as clear as it used to be’) and D12 (‘I find it easy to do the things I used to’). Psychometric research confirms that D11 and D12 serve as indicator variables for cognitive factors across diverse populations (Romera et al., 2008; Sakamoto et al., 1998). Furthermore, the strongest edge within this cluster occurred between occupational stress dimensions OS9 (I often feel annoyed about things around me) and OS10 (I feel irritable at times), a connection grounded in robust theoretical foundations. Irritability is defined as emotions of anger, agitation, and impatience (Saatchi, Olshansky, & Fortier, 2023). According to Barata et al., the most common symptom describing irritability is annoyance (Barata et al., 2016). Furthermore, within the occupational burnout cluster, EE was most strongly associated with cynicism. Theoretically, EE typically precedes cynicism (Maslach & Leiter, 2016). One study demonstrated a significant positive correlation between cynicism and EE (Verkuilen et al., 2020). Similarly, numerous studies have confirmed the developmental trajectory from EE to cynicism (Cordes & Dougherty, 1993; Kim et al., 2014; Leiter & Maslach, 1988).

EE was the most influential node within the OS–burnout–depression network. Within the occupational burnout–depression framework, multiple studies indicate that EE exhibits the highest expected impact among Chinese nursing populations (Wu et al., 2021; Zhang Y. et al., 2024; Zhang Z. et al., 2024). Physicians similarly emphasize the experience of ‘feeling exhausted after work’ within the EE dimension (Zhang Z. et al., 2024). Consistent with this, EE also exhibited significant predictive influence among clinical therapists (Gu et al., 2024). Following EE were OS11 (‘I feel low in mood’) and OS7 (‘I often feel depressed and unhappy because of work’), both nodes similarly reflecting emotional states – highlighting emotional distress as a common thread running throughout the network.

D18 (‘My life is pretty full’) represents another core symptom within the network. This finding is supported by Romera, Smith, Jones, Lee, and Brown (2008), who found that in a sample of 1049 MDD patients, feelings of emptiness (SDS item 18) exhibited the highest factor loading across all SDS items – underscoring its centrality in depressive symptomatology. This finding aligns with recent network analysis research, identifying emptiness as a core depressive symptom among teachers (Ma & Jia, 2024). Beyond identifying emptiness as a core depressive symptom, our network analysis further revealed D18’s pivotal role as a key hub within the integrated network of occupational stress, burnout, and depression.

The most critical bridging symptoms linking occupational stress to depression were ‘EE’, ‘CY’, and ‘PA’ within the burnout cluster, along with ‘OS11’ within the occupational stress cluster. Consistent with prior research, burnout emerged as a consequence of occupational stress and served as a full or partial mediating variable for subsequent depression (Ahola & Hakanen, 2007; Zhong et al., 2009). Building upon previous studies that often calculated overall scores, we found that each dimension of burnout consistently played a bridging role. Existing research has proposed a three-dimensional sequential model from occupational stress to burnout (Maslach & Leiter, 2016).

From a DAG perspective, CY and PA served as nodes connecting occupational stress to depression. Specifically, occupational stress symptoms triggered EE, which then activated CY; PA can be directly activated by other occupational stress symptoms and also activates CY. In other words, the concurrent reduced PA and EE jointly induced CY. These pathways align with structural theory principles (Edú-Valsania, Laguía, & Moriano, 2022), which emphasize the critical role of effective coping strategies in managing job stressors. Without effective coping strategies, the pathway from PA and EE to CY may emerge. Subsequently, both CY and reduced PA can independently trigger depression. Its upstream nodes are OS8 (‘I often feel fatigued’) and OS6 (‘I often have trouble sleeping because of work-related issues’), which may reflect an individual’s ineffective coping with occupational stressors in daily work.

Beyond the bridging effects of CY and PA on depression, occupational symptom OS11 (‘I feel low in mood’) exhibited a high BEI, directly influencing downstream depressive symptoms. This phenomenon involving high EI emotion-related nodes aligns with the emotion dysregulation model of emotion, which emphasizes that depression arises from negative emotional dysregulation coupled with diminished positive emotions (Hofmann, Sawyer, Fang, & Asnaani, 2012). Empirical studies indicate that negative affect is both associated with depression and serves as a risk factor for depression (Aldao, Nolen-Hoeksema, & Schweizer, 2010; Bos et al., 2013). A neuroimaging study demonstrates that the same amygdala–sACC connectivity, which regulates daily negative emotions, also increases during first depressive episodes, suggesting a shared neurobiological continuum between negative emotions and depression (Davey et al., 2015). Therefore, using Bayesian inference, we identified potential pathways leading to depression among Chinese healthcare workers and teachers, providing empirical support for both theoretical frameworks.

The DAG structure provides crucial guidance for identifying upstream intervention targets. Three occupational stress symptoms emerged as primary drivers: OS1 (I often worry about how to get the work done), OS7 (I often feel depressed and unhappy because of work), and OS8 (I often feel fatigued). In contrast, downstream symptoms – particularly depressive symptoms – appear to stem directly or indirectly from these upstream drivers. Proactive cognitive–behavioral strategies (e.g. planning and prevention) effectively alleviate stress (Epstein et al., 2024). Additionally, resilience factors such as a sense of humor and optimism serve as effective coping mechanisms (Tang, Raffone, & Wong, 2025).

This study has several limitations. First, although DAGs can examine potential causal relationships using cross-sectional data, longitudinal data are still required to validate these pathways. Second, the data were collected in Beijing, necessitating follow-up research in other regions. Third, the sample size is limited, and expanding the sample is urgently needed.

In summary, this study identified via network analysis four core symptoms: emotional exhaustion, life fulfillment, low mood, and frequent work-related depression/unhappiness; and four bridging symptoms: emotional exhaustion, low mood, cynicism, and personal accomplishment. Notably, emotional exhaustion and low mood serve dual roles as both core and bridging symptoms. The model also highlighted three source nodes, all tied to work-related stress and emotional reactions: worries about completing work, persistent exhaustion, and work-induced depression/unhappiness.

These findings reveal a clear pathway from work-related unhappiness, anxiety, and fatigue, through emotional exhaustion, diminished accomplishment, and cynicism, ultimately leading to depressive symptoms. Targeting these upstream and bridge symptoms in early intervention programs may help prevent the onset of burnout and depression.

## Supporting information

10.1017/S0033291726103547.sm001 Qiao et al. supplementary material Qiao et al. supplementary material

## Supplementary material

The supplementary material for this article can be found at http://doi.org/10.1017/S0033291726103547.
