# Occupational stress, burnout, and depression: an exploration from a network analysis perspective – ERRATUM

**DOI:** 10.1017/S0033291726105133

**Published:** 2026-08-10

**Authors:** Yiyuan Qiao, Haitao Xu, Yuling Li, Lei Shi, Shiqian Zhen, Shuchang He, XiangYang Zhang

When this article was published in Psychological Medicine it omitted to list Professors Shiqian Zhen and Shuchang He as co-corresponding authors alongside Professor XiangYang Zhang. This has since been updated.

The Publisher apologises for this error.
