# Supplementary material for: Occupational stress, burnout, and depression: an exploration from a network analysis perspective
Source: Psychol Med. 2026 Apr 27;56:e111. doi: 10.1017/S0033291726103547 (PMC13125937; doi:10.1017/S0033291726103547)

Supplementary Figure 1. Non-parametric bootstrap difference test for EI


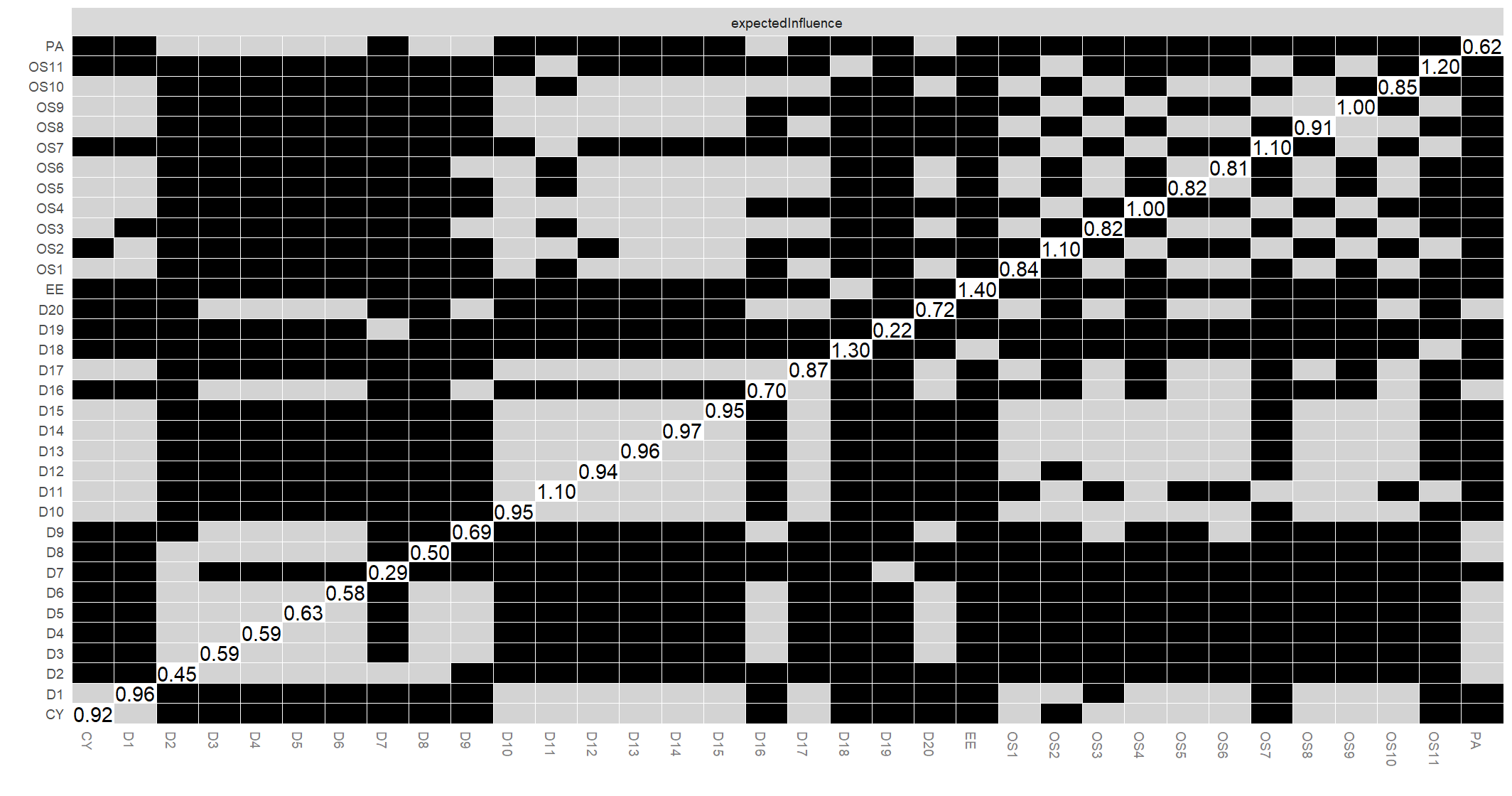


Note: Black boxes indicate a significance difference from corresponding edges while gray edges indicate no significant difference from corresponding edges at a significant level of 0.05.

Supplementary Fig. 2 95% confidence interval (CI) of the edge weights


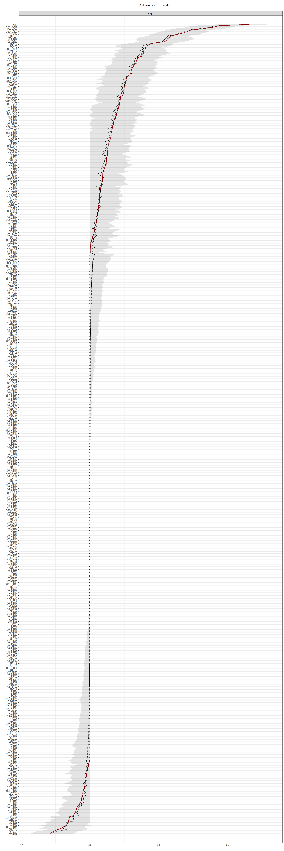


Supplementary Fig. 3 The CS-coefficient of EI and BEI


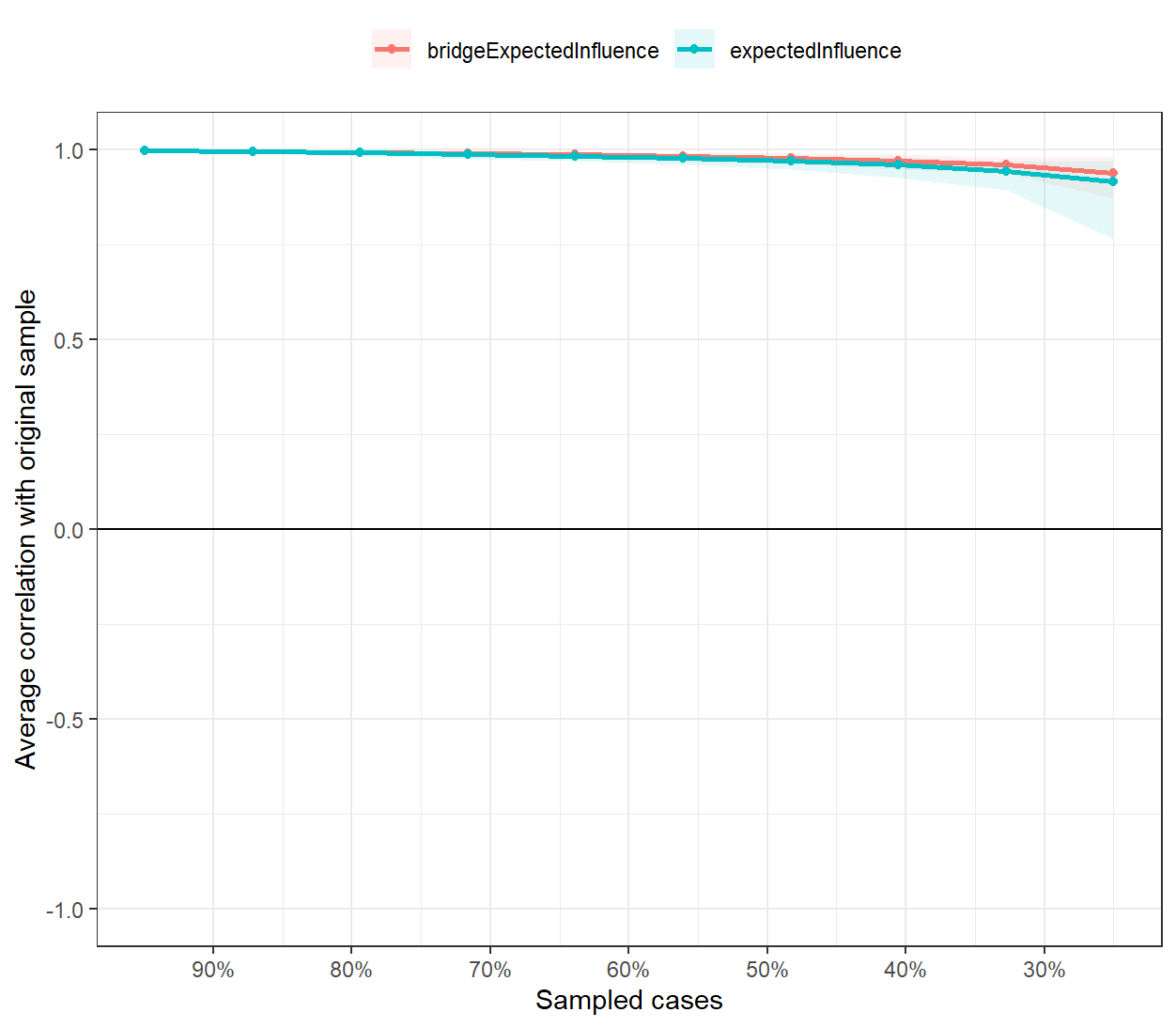

Supplement: Qiao et al. supplementary material [file S0033291726103547sup001.docx]
